# Giant Cell Tumor in the Distal End of the Ulna Managed by Darrach's Procedure: A Case Report

**DOI:** 10.7759/cureus.43101

**Published:** 2023-08-07

**Authors:** Madhavi M Kandarkar, Shivshankar Jadhav, Sanket M Kandarkar, Deepali S Patil

**Affiliations:** 1 Department of Musculoskeletal Physiotherapy, Ravi Nair College of Physiotherapy, Datta Meghe Institute of Higher Education and Research, Wardha, IND; 2 Department of Orthopaedic Surgery, Datta Meghe Institute of Higher Education and Research, Wardha, IND; 3 Department of Orthopaedic Surgery, Pravara Institute of Medical Sciences, Loni, IND

**Keywords:** darrach, phenol, physical therapy rehabilitation, distal ulna, giant cell tumors

## Abstract

Giant cell tumors (GCTs) are rare, benign, and locally invasive tumors, typically found in the epiphysis of long bones, most commonly at the distal femur and proximal tibia. To date, GCTs of the distal end of the ulna have been very rare. We document a case of a 38-year-old female with a distal ulna GCT, managed with en-bloc resection of the tumor with flexor carpi ulnaris and extensor carpi ulnaris tendon stabilization. The main aim of the GCT treatment is to prevent local recurrence and to maintain the function of the limb. Physical therapy was also given to the patient which helped in relieving pain, reducing edema, and increasing strength and range of motion. The patient was able to perform activities of daily living with the help of physical therapies and exercises. More research is needed to determine if broad excision of the distal ulna alone is a successful therapy for primary bone cancers affecting the distal ulna, including GCTs.

## Introduction

The tumor-bone giant cell tumor (GCT) is uncommon, often benign, and aggressive locally. It makes up between 3% and 5% of total primary bone malignancies. Adults aged 20 to 40 years are more frequently affected [1]. Neoplastic stromal cells, which make up the majority of proliferating cells, and mononuclear histiocytic cells with multinucleated giant cells that resemble osteoclasts are the pathophysiology's defining features [2]. Rarely do youngsters or people over the age of 65 acquire GCTs of the bone. One million people every year develop a GCT. The long bone meta-epiphysis is where the cancer is most commonly detected, notably at the distal end of the radius, femur, proximal humerus, and tibia. A rare location (0.45% to 3.2%) for a main bone GCT is the distal end of the ulna. Local recurrence after surgical therapy is the main problem with GCT treatment; it happens in 27.5% of patients after curettage alone, 12.27% when combined with adjuvants like a high-speed burr, phenol, polymethyl methacrylate, or liquid nitrogen, and 0% after en-bloc resection. We describe a 38-year-old woman who had a distal ulna GCT diagnosis. En-bloc excision of cancer along with stabilization of the flexor and extensor carpi ulnaris tendons was used as treatment. After a modified surgical technique for the excision of a GCT over the distal end of the ulna and stabilizing it with the extensor Carpi Ulnaris tendon, a full range of motion (ROM) was regained at the wrist joint, which distinguishes this case. Physical therapy can help patients improve their performance and quality of life after surgery. Muscle energy techniques (METs) are soft tissue release techniques used to treat a variety of musculoskeletal disorders, with the primary effects being the lengthening of a shortened or contracture of muscle and the expansion of a restricted joint's ROM [3].

## Case presentation

A 38-year-old female farmer had a complaint of swelling over her left wrist for the past six months. The patient claims to have fallen on her left wrist, after which she observed swelling over the wrist. The patient went to a traditional bone setter after complaining of excruciating pain in her left wrist following the incident. She was treated there using lape and massage techniques. Lape is prepared by mixing multiple ayurvedic extracts and it is applied over the swelling to get relief from pain and decrease the swelling. The patient was not satisfied with this as her pain and swelling did not subside. After one week, she reported to an orthopedic surgeon where she was examined and advised investigations like X-rays, computed tomography (CT), magnetic resonance imaging (MRI), and a Doppler test. The diagnosis of a GCT near the distal end of the ulna was made after these investigations.

**Figure 1:**
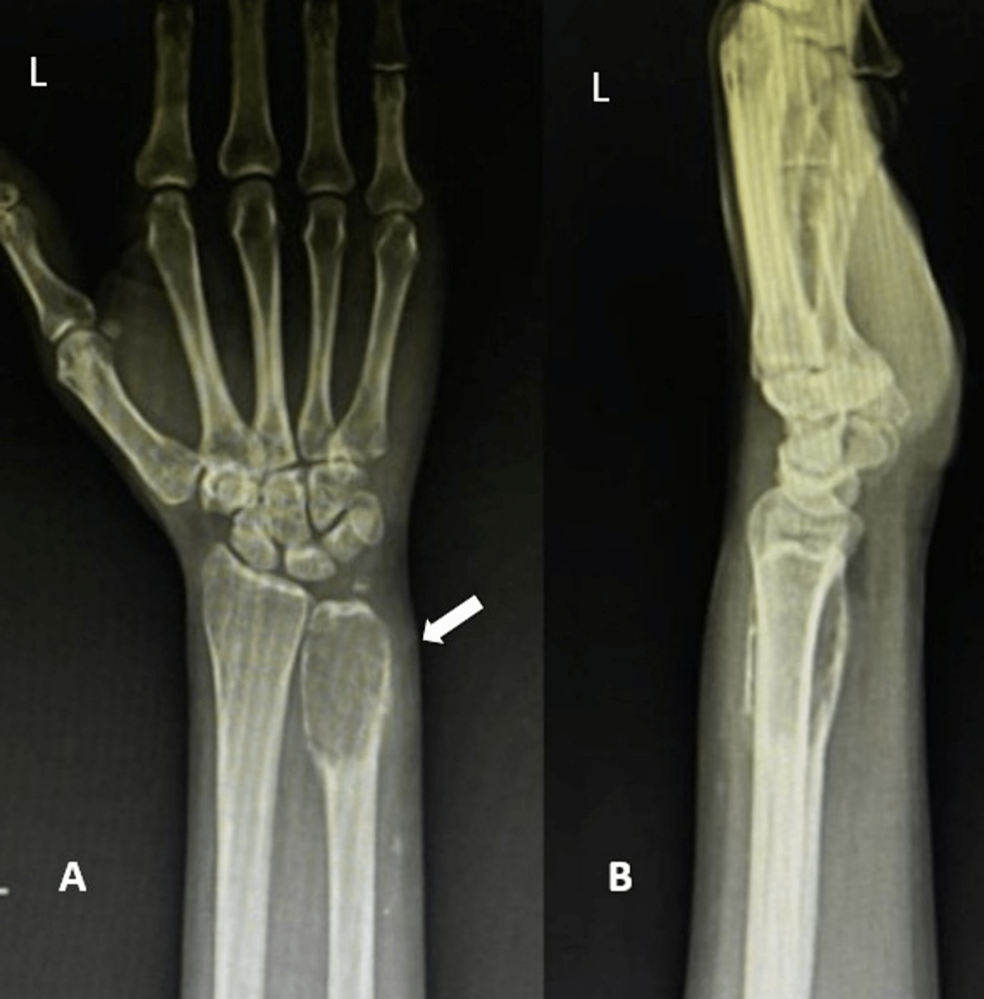
X-rays of the left wrist in AP and lateral views A and B show X-rays of the left wrist in anteroposterior and lateral views showing a radio-lucent and multi-lobar area with clear margins in the distal ulna (marked with an arrow). AP: Anteroposterior

The swelling was insidious in onset which gradually progressed to its current size. The patient's vital signs were stable and normal. After local examination, there was a visible mass of size 5.5 x 3 x 3 cm at the distal ulna region. The mass was immobile, hard, and painful on palpation with a visual analog score of 9 (normal range of VAS 0-10). The patient had limited left wrist movement due to pain. Severe disability is indicated by the PRWE (Patient-Rated Wrist Evaluation) and DASH (Disabilities of Arm, Shoulder, and Hand) scores of 46.7 and 44.64, respectively. The distal ulna was radiolucent and multi-lobar on radiography, with clean margins. No fracture was seen, although an osteolytic region was found at the left ulna (Figure 1). No signs of pulmonary metastases were visible on the chest X-ray. The wrist was examined and found to have painful pronation, supination, and circumduction, as well as a ROM of 40 degrees extension, 65 degrees flexion, 10 degrees radial deviation, and 5 degrees ulnar deviation. The opposing wrist's ROM was normal.

**Figure 2:**
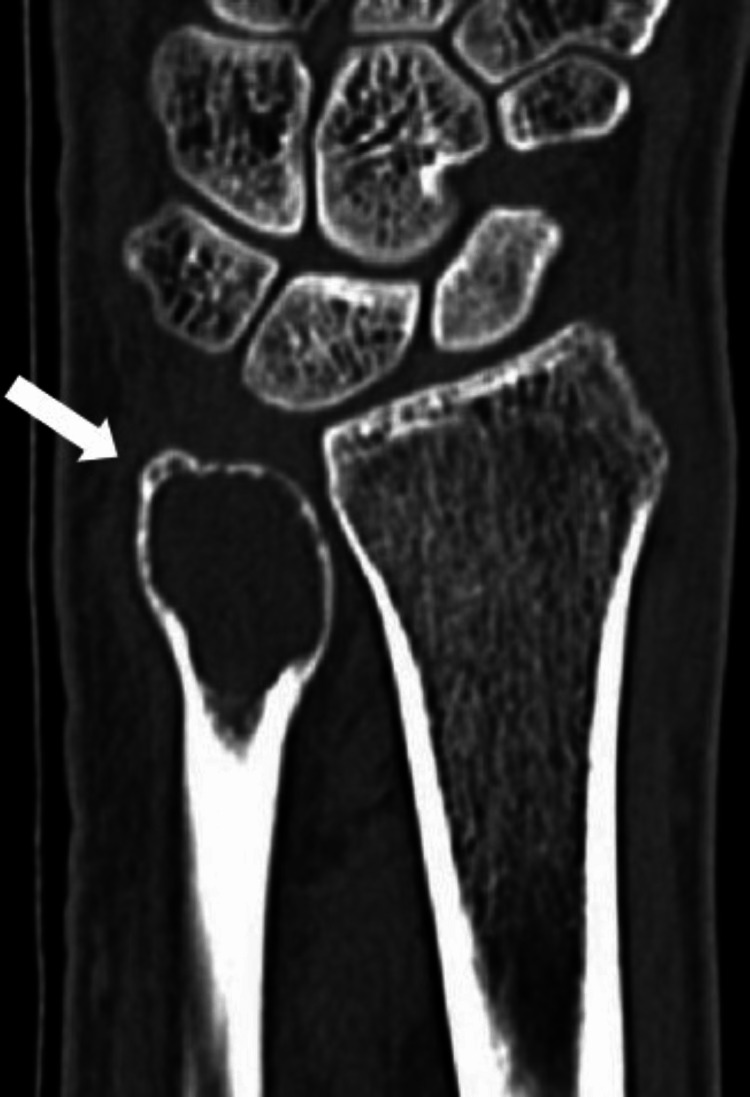
Computed tomogram of the left wrist CT scan of left wrist showing a thin and protruded cortex but no destruction of the cortex of the distal ulna

MRI investigation was carried out. The final impression reported a well-defined multiloculated expansile lytic lesion of size 19.4 x 19.1 x 36.2 mm involving the subarticular region of the ulna with a cortical breach. CT of the left wrist was also performed which showed a thin and protruded cortex but no evidence of destruction of the ulnar cortex (Figure 2).

**Figure 3:**
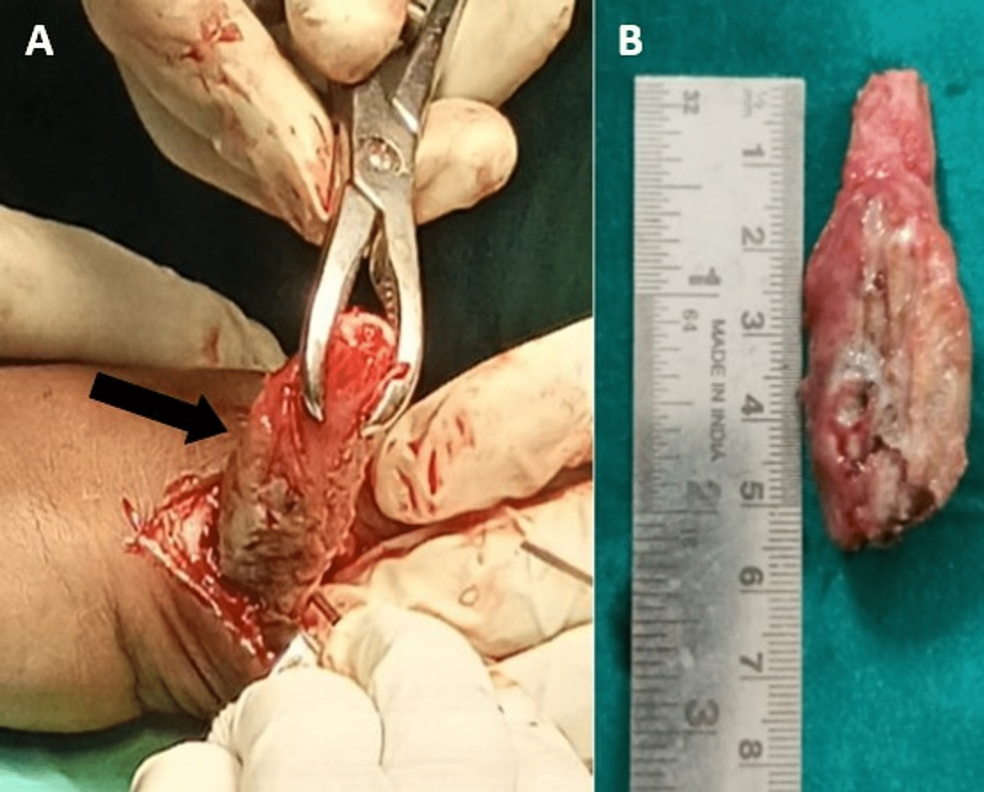
Intraoperative photograph of the resected tumor mass (marked with an arrow) A and B show intraoperative resected tumor mass with measurements.

The diagnosis of the GCT was made via an incisional biopsy. The lesion was categorized as stage II by the Enneking Classification for benign bone tumors. The damaged distal ulna was subsequently surgically excised en-bloc as part of the patient's treatment (Figure 3).

To assess bone consolidation, a clinical and radiographic follow-up was performed one, three, six, and twelve months after surgery. On the third postoperative day, the patient was referred to the physiotherapy department for hand rehabilitation. During the first three weeks, the patient was followed up and had reduced pain in the left wrist. The patient was able to perform all the movements. The PRWE and DASH score were 8 and 12.22 respectively which indicated mild disability. Every six months, chest radiographs were taken. Postoperatively, the patient was comfortable, and previous severe and continuous pain was relieved, except for surgical site pain, which was gradually diminishing. On the day of suture removal on postoperative day 14, the patient was pain-free. After three weeks of follow-up, physiotherapy was started, and the patient had continuously increasing mobility at the wrist joint, and at the sixth week, the patient recovered her wrist movements completely. After six months of surgery, the DASH score was 5.17. The PRWE score was 3 which is excellent. The patient can resume her normal daily activities and go about her daily life without any interruptions. Because the GCT has a high recurrence rate, the patient was encouraged to undergo routine examinations every six months to monitor for signs of recurrence.

The patient was treated surgically with en-bloc resection of the affected distal ulna by Darrach's procedure. Postoperatively, the patient was treated with antibiotics, antiplatelets, analgesics, and low-molecular-weight heparin. The patient's main post-operative complaints were dull-aching pain and swelling around the left wrist, with a numeric pain rating scale at rest rated 5/10, as well as difficulty in doing daily activities of living like gripping objects, grooming, combing, self-hygiene, and difficulties in occupation-related activities. For 10 weeks, the patient went to physiotherapy five days in a week. The physical therapy was as follows: 

In week 1, three sessions of paraffin wax bath for 20-30 minutes were given to relax muscles and reduce pain and edema. To prevent contractures and deformity, active shoulder ROM exercises, active assisted elbow ROM exercises, and passive forearm ROM exercises, including finger and thumb movements, were performed twice a day. Ten repetitions of these exercises were given. Thumb extensors and flexors, as well as finger extensors and flexors, were stretched with the forearm in a semi-prone position. The stretch force was applied for 20-30 seconds five times a day. Further for another three days, cryotherapy was used to reduce swelling.

In weeks 2- 4, as the pain and swelling subsided, the cryotherapy was stopped. The paraffin wax therapy was continued, but the duration was reduced due to the significant reduction in swelling. The ROM exercises were continued, but the repetitions were reduced to 20 per day. Gradual joint mobilization began with 30-second doses of Maitland grade I and II, followed by 3-5 minutes of stretching. To improve and facilitate opposition, the patient was given a finger spring exerciser with the least resistance. The patient was given a MET post isometric relaxation type in which a submaximal contraction of the hypertonic muscle away from the barrier was performed between 5 and 10 seconds and resistance was applied in the opposite direction with inhalation and after the contraction.

In weeks 5-8, the exercises for ROM were continued. Gradually increasing levels of patient response-based stretching and progressive mobilization were used in grades III and IV. To enhance strength, isometric shoulder, and elbow strengthening exercises were started with a 10-second hold and 10 repetitions of each. The patient was instructed to commence flexion, extension, supination, and pronation while applying resistance and holding it for 10 seconds by the therapist as a means to encourage the patient.

In weeks 8-10, to improve and strengthen the opponens muscle, a finger spring exerciser with a high resistance level was given. The gel ball exerciser began to aid in grip. Elbow flexor static stretching and ROM exercises were performed actively. Static stretching of the thumb and finger extensors and flexors was maintained to stimulate the Golgi tendon organ and to prevent contracture. Functional task-oriented exercises were implemented to make daily activities easier.

Home program 

The patient was given a home exercise program to follow after the physiotherapy session. The patient was instructed to perform rubber band exercises at home. The patient's thumb and index finger were wrapped in a rubber band, and she was instructed to stretch it to improve index finger and thumb extension as well as thumb abduction. To improve grip strength, the patient was told to use a sponge ball at home. The patient was instructed to follow up in the next 15 days.

## Discussion

GCTs of the bone, rare benign neoplasms with locally aggressive activity, make up around 5% of all primary bone tumors. There are 0.65 to 1 incidence per million individuals yearly, according to reports. According to studies, this ailment primarily affects people between the ages of 20 and 40, and women are more affected than men. Cooper and Travers published the first description of the bone GCT in 1818. Nelaton and Virchow have drawn attention to its territorial hostility and its propensity for malignancy. Although an uncommon, mostly benign tumor, it may act in an unexpected way independent of the findings of histological or radiological tests. Larger tumors can spread into the metaphysis but may also affect the diaphysis. It is most commonly found in meta-epiphysis of long bones and involves the subchondral bone without affecting the articular surface. The proximal end of the tibia, humerus, distal end of the femur, and radius are all common locations. Between 3% and 5% of all bone tumors are GCTs, and 21% of benign bone tumors are GCTs [4]. It affects women in their third to fourth decades of life in 70% of instances. Only 0.45% to 3.2% of all primary bone GCTs occur at the distal epiphysis of the ulna, making it an uncommon location for a primary bone GCT. In the past, amputation or extensive resections along with secondary reconstructions were used to treat these malignancies. Table 1 shows various surgical treatments available for the treatment of GCTs [1].

**Table 1:** Available surgical procedures

Available Surgical Procedures
Intralesional curettage
Curettage and bone grafting
Cryotherapy of the cavity after curettage
Application of phenol after curettage
Radiation
Insertion of methyl methacrylate cement in the cavity after curettage
Resection followed by allograft
En-bloc resection with or without reconstruction or stabilization of the ulna and the prosthetics used

The variables such as location, cortical bone destruction, biological activity, and evidence of pathological fracture are related to the tumor and determine the modality of treatment [4]. Although the tumor can be managed with en-bloc resection that significantly reduces the risk of recurrence, the functional outcome is not that good. An excellent functional outcome can be achieved by simple curettage, but the recurrence rate is much higher approximately 40% as compared to the patient who received adjuvant therapy [5]. In addition to curettage, other adjuvant treatments have been used intraoperatively, such as phenol, cryotherapy, and polymethyl methacrylate or cement. When cement is employed, the recurrence rates vary from 6% to 8%, and following cryosurgery, they are around 2.3% [6].

The Canadian Sarcoma Group carried out multi-center research and found that the use of any adjuvant therapy or the filling material had no absolute influence on the recurrence, which was reported to be 17% overall [7]. Schajowicz reported in his study that curettage alone is not a useful oncological procedure, but when combined with adjuvant therapy, it provides an excellent functional result concerning one-block excision [8]. Therefore, the goal of treatment is to achieve a balance between the restoration of the skeletal segment functionally and oncological radically [9]. The distal ulna helps the forearm rotate. Moreover, it supports grip power and preserves the connection between the carpus and the distal end of the radius. The GCT and other benign aggressive distal ulna tumors can be treated using one of two methods. When the GCT is restricted to the bone cortex, curettage may be utilized as the initial form of therapy. Another alternative is distal ulna resection, which is done when the tumor is not contained inside the cortex when there is a soft tissue component, when there is a pathological fracture, or when a prior operation was unsuccessful [10]. According to Darrach, the distal ulna may be removed while compromising function, and he suggested doing so for degenerative conditions [11].

Without utilizing any biological rebuilding techniques, such as bone grafting, the distal end of the ulna was removed in our instance. Within three weeks of the procedure, our patient was able to perform her wrist movements without any pain. Three weeks postoperative, the DASH score was 12.22, which indicates a mild disability. This outcome was in line with research conducted by Cooney et al. that reported a 75% good outcome following distal ulnar GCT excision [12]. The patient visited our outpatient clinic six months following surgery with a favorable outcome and no indication of ulnar nerve palsy or restriction of forearm function. Six months following the operation, the final DASH score was 5.17, a substantial improvement from the baseline DASH score of 44.64 (severe disability). After the six-month follow-up, the PRWE in our patient was outstanding. This shows that there were no appreciable wrist disturbances during routine activities. Evaluations of pain and function were excellent.

Painful stump or click instability may result as a complication to excessive resection of the stump. In our case, appropriate identification of the margins of the tumor and measurement of the area of the excision was important. Thus, with better precision and planning good functionality of the forearm can be achieved. Additionally, postoperative physical therapy helped the patient regain the use of their hand for everyday tasks by strengthening the hand muscles and power grip. By combining creep and plastic change in connective tissue, it is hypothesized that MET can relax articular restrictions, lengthen muscle fibers, and enhance the ROM [13]. The paraffin wax bath is beneficial for promoting blood flow and relaxing the muscles [14].

## Conclusions

Given that the DASH score improved between three and six weeks of the surgery in our patient, en-bloc resection alone may become to be the preferred management for GCT of the distal ulna. The patient was given a comprehensive recovery physiotherapy rehabilitation plan following surgery, which helped her relieve pain, reduce edema, and increase strength and ROM. The patient was able to perform daily living tasks with the help of routine exercises. More research is needed to determine if broad excision of the distal ulna alone is a successful therapy for primary bone cancers affecting the distal ulna, including GCTs.
